# Responding to avian influenza in poultry farms in Victoria, Australia

**DOI:** 10.2471/BLT.24.292748

**Published:** 2025-05-27

**Authors:** Karen Blaney, Luke Cardamone, Naomi E Clarke, Mark J Hayes, Michael Muleme, Bridgette J McNamara, Edura Jalil, Storm Holwill, Chuan Kok Lim, Helen O’Brien, Elly Layton, Alexander Fidao, Sally Salmon, Rebecca Kinnear, Mark Ford, Mohammad Akhtar Hussain, Eugene Athan

**Affiliations:** a Barwon South West Public Health Unit, Barwon Health, University Hospital Geelong, PO Box 281, Geelong 3220, Victoria, Australia.; b Department of Infectious Diseases, Barwon Health, Geelong, Australia.; c South East Public Health Unit, Monash Health, Melbourne, Australia.; d Western Public Health Unit, Western Health, Melbourne, Australia.; e Victorian Infectious Diseases Reference Laboratory, Royal Melbourne Hospital, Melbourne, Australia.; f Victorian Department of Health, Melbourne, Australia.; g Agriculture Victoria, Department of Energy, Environment and Climate Action, Leongatha, Australia.; h Commonwealth Scientific and Industrial Research Organisation, Australian Centre for Disease Preparedness, Geelong, Australia.

## Abstract

**Objective:**

To describe a multi-agency public response to an outbreak of avian influenza virus in poultry farms in Victoria, Australia, in 2024.

**Methods:**

After detecting an outbreak of high-pathogenicity avian influenza at a poultry farm and notifying the Victorian health department, Agriculture Victoria identified a further seven infected premises through tracing and surveillance activities. Testing at the Australian Centre for Disease Preparedness identified high-pathogenicity H7N3 at seven premises in the Golden Plains Shire, and high-pathogenicity H7N9 at a property in the Terang region in the Corangamite Shire. The Victorian health department established a multi-agency incident management team, and we defined, identified and managed contacts and suspected cases. Public health actions included influenza vaccination, antiviral prophylaxis, active surveillance of high-risk contacts, and supporting infection prevention and control measures.

**Findings:**

We identified a total of 212 (165 high- and 47 low-risk) unique human contacts with residence locations spread across 25 local government areas. We identified 20 suspected cases from six of the eight infected premises, all of whom tested negative for influenza A. Of the 172 unique high-risk contacts and suspected cases, local health services and clinics reported that 19.2% (33) received antiviral medication and 27.3% (47) received the seasonal influenza vaccine.

**Conclusion:**

Our rapid, coordinated, multi-agency response was a success; however, governments, agricultural industries and health workers must strengthen preparedness and response strategies across national, state, regional and local levels to improve surveillance, foster collaboration and address gaps in preventive health care.

## Résumé

**Objectif:**

Décrire la réaction publique de plusieurs agences gouvernementales à un foyer de grippe aviaire dans des élevages de volailles de l’État de Victoria, en Australie, en 2024.

**Méthodes:**

Après avoir détecté un foyer de grippe aviaire hautement pathogène dans un élevage de volailles et averti le ministère de la Santé de l’État de Victoria, le ministère de l’Agriculture a identifié sept autres élevages infectés grâce à des activités de surveillance. Les tests effectués par le Centre australien de préparation aux maladies ont permis d’identifier le virus H7N3 hautement pathogène sur sept sites du comté de Golden Plains, et le virus H7N9 hautement pathogène dans une propriété de la région de Terang, dans le comté de Corangamite. Le ministère de la Santé de l’État de Victoria a mis en place une équipe de gestion des incidents répartie sur plusieurs agences, et nous avons défini, identifié et géré les contacts et les cas suspects. Parmi les mesures de santé publique figuraient la vaccination contre la grippe, la prophylaxie antivirale, la surveillance active des contacts à haut risque et le soutien des mesures de prévention et de contrôle des infections.

**Résultats:**

Nous avons identifié un total de 212 contacts humains uniques (165 à haut risque et 47 à faible risque) dont les lieux de résidence sont répartis dans 25 divisions gouvernementales. Nous avons identifié 20 cas suspects dans six des huit sites infectés, tous testés négatifs pour la grippe A. Sur les 172 contacts uniques à haut risque et cas suspects, les cliniques et services de santé locaux ont rapporté que 19,2% (33) ont reçu des médicaments antiviraux et 27,3% (47) ont reçu le vaccin contre la grippe saisonnière.

**Conclusion:**

Notre réaction rapide et coordonnée entre plusieurs agences a remporté un franc succès. Cependant, les pouvoirs publics, les industries agricoles et les professionnels de la santé doivent renforcer les stratégies de préparation et de réactions aux niveaux national, étatique, régional et local afin d’améliorer la surveillance, d’encourager la collaboration et de combler les lacunes en matière de soins de santé préventifs.

## Resumen

**Objetivo:**

Describir la respuesta pública interinstitucional ante un brote de virus de la gripe aviar en granjas avícolas del estado de Victoria, Australia, en 2024.

**Métodos:**

Tras la detección de un brote de gripe aviar de alta patogenicidad en una granja avícola y la notificación al departamento de salud de Victoria, Agriculture Victoria identificó otras siete instalaciones infectadas mediante actividades de rastreo y vigilancia. Las pruebas realizadas por el Centro Australiano para la Preparación de Enfermedades confirmaron la presencia del subtipo H7N3 de alta patogenicidad en siete instalaciones del distrito de Golden Plains y del subtipo H7N9 de alta patogenicidad en una propiedad ubicada en la región de Terang, distrito de Corangamite. El departamento de salud de Victoria estableció un equipo de gestión de incidentes interinstitucional. Se definieron, identificaron y gestionaron los contactos y los casos sospechosos. Las medidas de salud pública incluyeron vacunación antigripal, profilaxis antiviral, vigilancia activa de contactos de alto riesgo y apoyo a las medidas de prevención y control de infecciones.

**Resultados:**

Se identificaron 212 contactos humanos únicos (165 de alto riesgo y 47 de bajo riesgo), residentes en 25 áreas de gobierno local. Se identificaron 20 casos sospechosos vinculados a seis de las ocho instalaciones infectadas; todos dieron negativo para gripe A. Entre los 172 contactos únicos de alto riesgo y casos sospechosos, los servicios de salud locales y clínicas notificaron que el 19,2% (33) recibió medicación antiviral y el 27,3% (47) recibió la vacuna antigripal estacional.

**Conclusión:**

La respuesta rápida, coordinada e interinstitucional fue eficaz; sin embargo, los gobiernos, el sector agropecuario y el personal sanitario deben reforzar las estrategias de preparación y respuesta en todos los niveles —nacional, estatal, regional y local— para mejorar la vigilancia, fomentar la colaboración y subsanar las deficiencias en la atención preventiva.

## ملخص

الغرض وصف استجابة عامة متعددة الجهات لتفشي فيروس إنفلونزا الطيور في مزارع الدواجن في فيكتوريا، أستراليا، عام 2024.

الطريقة بعد اكتشاف تفشي فيروس إنفلونزا الطيور شديد الإمراضية في مزرعة دواجن، وإبلاغ وزارة الصحة في فيكتوريا، حددت وزارة الزراعة في فيكتوريا سبعة مواقع أخرى مصابة من خلال أنشطة التتبع والمراقبة. كشفت الاختبارات التي أجراها "المركز الأسترالي للتأهب للأمراض" عن فيروس H7N3 شديد الإمراضية في سبعة مواقع في مقاطعة جولدن بلينز، وفيروس H7N9 شديد الإمراضية في عقار بمنطقة تيرانج في مقاطعة كورانجاميت. أسست وزارة الصحة في فيكتوريا فريقًا متعدد الهيئات لإدارة الحوادث، وقمنا بتحديد المخالطين والحالات المشتبه بها وتحديدها وإدارتها. وشملت إجراءات الصحة العامة التطعيم ضد الإنفلونزا، والعلاج الوقائي بمضادات الفيروسات، والمراقبة النشطة للمخالطين المعرضين لخطر كبير، ودعم إجراءات الوقاية من العدوى ومكافحتها.

النتائج حددنا ما مجموعه 212 جهة اتصال بشرية فريدة (165 عالية الخطورة و47 منخفضة الخطورة)، مع أماكن إقامة موزعة على 25 منطقة حكومية محلية. حددنا 20 حالة مشتبه بها من ستة من المباني الثمانية المصابة، وجميعها جاءت نتائج اختباراتهم سلبية للإنفلونزا أ. من بين 172 جهة اتصال فريدة عالية الخطورة وحالات مشتبه بها، أفادت الخدمات الصحية والعيادات المحلية أن 19.2% (33) تلقت أدوية مضادة للفيروسات، و27.3% (47) تلقت لقاح الإنفلونزا الموسمية.

الاستنتاج كانت استجابتنا السريعة والمنسقة ومتعددة الهيئات ناجحة؛ ومع ذلك، يجب على الحكومات والصناعات الزراعية والعاملين في مجال الصحة تعزيز استراتيجيات التأهب والاستجابة على المستويات الوطنية والولائية والإقليمية والمحلية، لتحسين المراقبة وتعزيز التعاون ومعالجة الثغرات في الرعاية الصحية الوقائية.

## 摘要

**目的:**

旨在描述针对 2024 年澳大利亚维多利亚州家禽场爆发的禽流感病毒疫情采取的多机构公共应对措施。

**方法:**

在检测到某一家禽场爆发了高致病性禽流感疫情并报告维多利亚州卫生部之后，维多利亚州农业局通过追溯和监测行动进一步确定了七处发生疫情的场所。经澳大利亚疾病预防中心检测后确认，在黄金平原郡有七处场所出现了高致病性 H7N3 病毒，且在科兰加迈特郡特兰地区也有一家农场出现了高致病性 H7N9 病毒。维多利亚州卫生部成立了一个多机构事件管理小组，我们则负责对接触者和疑似病例进行界定、确认和管理。采取的公共卫生行动包括呼吁民众接种流感疫苗和接受抗病毒预防治疗、积极监测高风险接触者的身体状况以及采取支持性感染预防和控制措施。

**结果:**

我们总共确定了 212 名（165 名高风险接触者和 47 名低风险接触者）需要特别关注的人类接触者，这些接触者的住所分散在 25 个地方政府辖区内。在八处出现疫情的场所中，我们在六处场所中确定了 20 名疑似病例，且所有疑似病例的甲型流感检测结果均为阴性。据当地卫生服务机构和诊所报告，在这 172 名需要特别关注的高风险接触者和疑似病例中，19.2%（33 名）接受过抗病毒药物治疗，且 27.3%（47 名）接种过季节性流感疫苗。

**结论:**

我们的快速、多机构协同应对行动颇见成效；但是，各级政府、农业产业部门以及卫生工作者都必须从国家、州、地区和地方层面强化防范和应对策略，从而提升监测能力、推动各方合作并解决在预防性卫生保健方面存在的问题。

## Резюме

**Цель:**

Описать реакцию общественности с участием различных органов власти на вспышку вирусного птичьего гриппа, охватившую птицефермы штата Виктория в Австралии в 2024 году.

**Методы:**

После выявления вспышки высокопатогенного вируса птичьего гриппа на птицеферме и уведомления Министерства здравоохранения штата Виктория Комитет развития сельского хозяйства штата Виктория (Agriculture Victoria) выявил еще семь зараженных объектов, используя процедуры отслеживания и надзора. Анализ, выполненный Австралийским центром готовности к борьбе с заболеваниями (Australian Centre for Disease Preparedness), выявил высокопатогенный штамм H7N3 на семи объектах в графстве Голден-Плейнс, а также высокопатогенный штамм H7N9 на объекте в районе Теранг графства Корангамайт. Министерство здравоохранения штата Виктория создало группу реагирования из представителей различных органов власти, которая занималась определением и выявлением контактов и случаев возможной инфекции, а также управлением ими. Противоэпидемические мероприятия включали вакцинацию от гриппа, профилактический прием противовирусных препаратов, активный надзор за контактными лицами из групп высокого риска, а также усиление мер по профилактике и контролю инфекций.

**Результаты:**

Авторы выявили в общей сложности 212 уникальных случаев контакта людей с инфекцией (165 высокого риска и 47 низкого), проживающих в 25 местных административных районах. Было выявлено 20 случаев возможной инфекции на шести из восьми зараженных объектов, и все они оказались отрицательными по гриппу А. По данным местных органов здравоохранения и клиник, из 172 уникальных эпизодов контакта с высоким риском и случаев возможной инфекции в 19,2% случаев (у 33 человек) было предоставлено лечение противовирусными препаратами, а в 27,3% случаев (у 47 человек) была введена сезонная вакцина от гриппа.

**Вывод:**

Быстрый и скоординированный ответ со стороны разных органов власти и общественности был успешным, однако правительствам, сельскохозяйственным учреждениям и работникам сферы здравоохранения следует повысить подготовленность и улучшить стратегии реагирования на всех уровнях (национальном, на уровне штата, в регионах и на местах), чтобы укрепить надзор, содействовать сотрудничеству и ликвидировать упущения в вопросах медицинской профилактики заболеваний.

## Introduction

Although strict biosecurity measures in Australia have limited the emergence of many zoonotic diseases, the presence of migratory birds means that the threat of avian influenza viruses cannot be eliminated. Because of the occasional transmission of these viruses to humans, they represent a threat to global public health.^1^^–^^3^ Human cases of avian influenza have a risk of severe disease and the potential for co-infection with seasonal and avian influenza, increasing the risk of genetic reassortment and emergence of a potentially pandemic subtype.

There exist global concerns about the spread of avian influenza viruses, of which subtypes H5 and H7 are classified as either low- or high-pathogenicity according to disease severity in poultry.^4^ Because Australia remains the only continent free from high-pathogenicity subtype H5N1, this subtype (especially H5N1 clade 2.3.4.4b) is of particular concern.^5^ Studies have shown higher seroprevalence of H7N9 and H7N3 among poultry workers in endemic areas compared with the general population.^6^^–^^8^ In 2004, two people with avian influenza virus were linked to a H7N3 poultry outbreak in Canada.^9^ Subtype H7 viruses have caused at least a further 1600 reported human infections, most of which were associated with the zoonotic H7N9 strain that emerged in China in 2013.^10^ Although mortality in humans with H7N9 reached an alarming 39.2% (615/1567), human infections with other H7 low- and high-pathogenicity strains have mostly been mild and linked to direct contact with infected poultry.^10^ Human-to-human transmission of avian influenza is rare, with probable limited cases associated with high-pathogenicity H5N1 and H7N9 and low-pathogenicity H7N9 infections, and in settings of prolonged close contact with the index case.^4^

A large outbreak of high-pathogenicity H7N3 and H7N9 occurred among poultry farms in Victoria, Australia, during May–June 2024. Before 2024, high-pathogenicity H7 virus was last reported in domestic poultry in Victoria in 2020. The Australian Centre for Disease Preparedness reported that the H7N9 strain detected during this outbreak differs genomically from the strain found in Asia in 2013.

Victoria has well-established poultry meat and egg industries, operating under stringent biosecurity guidelines.^11^^–^^13^ Approximately 3.5 million birds produce 66 million dozen eggs per year,^11^ accounting for approximately one fifth of Australia’s total egg production;^14^ avian influenza outbreaks can therefore have profound implications for the poultry industry. Control measures, including the depopulation of farms, can lead to financial loss for the poultry sector and have wider economic implications.^15^ The Australian government provides national coordination of emergency animal disease response activities, although individual states and territories are responsible for delivering these responses. Nationally agreed governance arrangements are described in reports by Animal Health Australia.^16^^,^^17^ In Victoria, the legal support for emergency animal disease responses is provided by the Livestock disease control act (1994),^18^ regulating quarantine of affected properties, animal and product movement restrictions, and compensation to farmers.

In this report, we describe the management of people exposed to avian influenza in an outbreak setting, as well as the control measures implemented to prevent further transmission. We describe how a successful collaboration between key agencies – the Victorian Department of Health, public health units, the Victorian Infectious Diseases Reference Laboratory and the Australian Centre for Disease Preparedness – facilitated a rapid and effective public health response.

## Methods

### Tracing and surveillance

Agriculture Victoria, a branch of the Victorian Department of Energy, Environment and Climate Action, notified the Victorian Department of Health of a suspected outbreak of high-pathogenicity avian influenza at a poultry farm near Meredith in the Golden Plains Shire, Victoria, on 21 May 2024.^19^ After launching tracing and enhanced surveillance activities, Agriculture Victoria identified a further seven infected premises over the following 4-week period.

From the eight infected premises (three mixed free-range and cage egg layer poultry farms; two free-range egg layer poultry farms; one pullet-rearing barn-style farm; one indoor barn-layer farm; and one duck egg and meat farm), testing at the Australian Centre for Disease Preparedness identified high-pathogenicity H7N3 at seven premises in the Meredith region of the Golden Plains Shire, and high-pathogenicity H7N9 at a property in the Terang region in the Corangamite Shire (Table 1 and online repository).^20^ The Australian Centre for Disease Preparedness reported that the H7N3 strain detected was genetically consistent across the seven premises, suggesting farm-to-farm transmission rather than independent introduction events.

**Table 1:** Dates of onset of avian influenza virus and identification of infected premises, with the number of high- and low-risk contacts identified at each, at eight sites in Victoria, Australia

Infected premises identifier^a^	Strain	Onset date of disease in poultry	Date infected premises identified	No. identified contacts (*n* = 212)^b^		
				High-risk (total: 165)	Low-risk (total: 47)	Total per site
IP1	H7N3	17 May2024	22 May 2024	79	18	97
IP2	H7N9	22 May 2024	23 May 2024	24	6	30
IP3a^c^	H7N3	2 June 2024	2 June 2024	8	7	15
IP4	H7N3	1 June 2024	5 June 2024	4	1	5
IP5	H7N3	1 June 2024	7 June 2024	8	8	16
IP3b^c^	H7N3	9 June 2024	12 June 2024	20	8	28
IP6	H7N3	12 June 2024	13 June 2024	2	2	4
IP7	H7N3	13 June 2024	17 June 2024	15	3	18
IP8	H7N3	20 June 2024	25 June 2024	7	2	9

^a^ Infected premises numbered according to date on which clinical signs became evident in poultry.

^b^ Number of unique contacts (some contacts were linked to more than one premises).

^c^ Neighbouring sites owned by the same company; when H7N3 was detected at the second site, the infected premises became referred to as IP3a and IP3b.

Agriculture Victoria immediately imposed quarantine under section 110 of the Livestock disease control act 1994, followed by depopulation (the killing of infected and in-contact poultry) and decontamination (the process of clearing the farm of infection).^21^ Agriculture Victoria also enforced a 5 km radius restriction zone and a 20 km radius control area buffer zone around all infected premises;^21^ only permitted the movement of poultry, products or equipment under permit;^22^ introduced the requirement that all poultry in defined areas were housed to prevent further spillover events from wild birds (only removing this requirement when extensive testing demonstrated a significantly reduced viral burden in the affected areas); and established an intensive surveillance programme in all restriction zones,^21^ which included the testing of dead birds, the collection of production data from commercial flocks, contact with bird owners to check on bird health and the testing of birds to allow permitted movements. The online repository contains further information on surveillance and tracing activities led by Agriculture Victoria.^20^

### Outbreak response

The Victorian health department acted as a supporting agency for the overall outbreak response and led the human health response, establishing an incident management team on 22 May 2024 with representatives from public health units, the Victorian Infectious Diseases Reference Laboratory and Agriculture Victoria. The Victorian health department conducted online incident management team meetings using Teams (Microsoft, Redmond, United States of America) and distributed situational reports by email.

Barwon South West Public Health Unit led the operational response for six of the infected premises; Western Public Health Unit led the operational response for the remaining two premises. At a regional level, the public health units coordinated the response in collaboration with local stakeholders including health services, pharmacies, general practitioners and the Western Victoria Primary Health Network.

As the overall control agency, Agriculture Victoria chaired regular meetings with key stakeholders, including a Victorian health department representative. Agriculture Victoria also established an incident control centre locally, with an Emergency management liaison officer representing the Victorian health department. 

### Contacts and cases

#### Definitions

To define and manage our incident contacts and cases, we adopted the 2016 Communicable Diseases Network Australia series of national guidelines for avian influenza in humans,^23^ conducted a rapid literature review and consulted international guidelines, including those published by the United States Centers for Disease Control and Prevention.^24^ Although our literature review provided relatively limited information about the risk to humans from high-pathogenicity H7N3 and H7N9 strains in poultry, we developed contact (Box 1) and case (Box 2) definitions using the other available guidelines.

Box 1 **Definitions of contacts in management of response to outbreak of avian influenza, Victoria, Australia, 2024**

**Contact**

Someone who attended an infected premises from the beginning of the risk period.^a^

**High-risk contact**

Someone who attended an infected premises during the risk period^a^ and had close or direct contact with live or deceased birds, faeces, litter or eggs without full personal protective equipment.

**Low-risk contact**

Someone who attended an infected premises during the risk period^a^ but did not have close or direct contact with live or deceased birds, faeces, litter or eggs.

**Not a contact**

Someone who attended an infected premises during the risk period^a^ while wearing full personal protective equipment and having received personal protective equipment usage training.

^a^ The risk period is defined as the 10-day period before the onset of symptoms in poultry.

Box 2 **Definitions of human cases in management of response to outbreak of avian influenza, Victoria, Australia, 2024**

**Confirmed case**

Laboratory-definitive evidence^a^ and clinical evidence^b^

**Probable case**

Laboratory-suggestive evidence,^a^ clinical evidence and epidemiological evidence^c^

**Suspected case**

Clinical evidence^b^ and epidemiological evidence^c^

^a^ See online repository for full case definitions, including laboratory-definitive and laboratory-suggestive evidence.^20^^b^ Clinical evidence refers to an acute illness characterized by: (i) fever (temperature > 38 °C), or history of fever plus one or more of: cough, rhinorrhoea, myalgia, headache, dyspnoea and diarrhoea; (ii) conjunctivitis; or (iii) infiltrates or evidence of an acute pneumonia on chest radiograph plus evidence of acute respiratory insufficiency (hypoxaemia, severe tachypnoea).^c^ Epidemiological evidence refers to one or more of the following exposures during the risk period: (i) close contact (within 1 m) with a person (e.g. caring for, speaking with or touching) who is a probable or confirmed case; (ii) exposure (e.g. handling, slaughtering, defeathering, butchering, preparation for consumption) to poultry or wild birds or their remains or to environments contaminated by their faeces in an area where avian influenza infections in animals or humans have been suspected or confirmed in the last month; (iii) consumption of raw or undercooked poultry products in an area where avian influenza infections in animals or humans have been suspected or confirmed in the last month; (iv) close contact with a confirmed infected animal other than poultry or wild birds (e.g. cat or pig); or (v) handling samples (animal or human) suspected of containing avian influenza virus in a laboratory or other setting.

#### Contact identification

Agriculture Victoria notified the Victorian health department of each infected premises. This information was shared with the relevant public health unit, who requested a list of workers, visitors and contractors that had attended the premises during the risk period (defined as commencing 10 days before the onset of symptoms in the poultry). The public health unit staff interviewed each contact to collect demographic information, complete an exposure assessment (e.g. occupational role, level of poultry exposure, use of personal protective equipment and influenza vaccination status), and determine whether they had current or recent influenza-like symptoms.

#### Contact management

We classified and managed contacts according to their risk level (high or low; Box 1). We prescribed 75 mg of oseltamivir (provided free of charge by local health services) per day for 10 days,^25^ if within 7 days of last unprotected exposure, for high-risk contacts. We also offered an influenza vaccination to high-risk contacts who had not already received an influenza vaccine for the 2024 influenza season, in accordance with *Australian Immunisation Handbook* recommendations.^26^ We set up dedicated vaccine clinics in the Barwon South West region, and contacts could also attend routine influenza vaccine clinics free of charge. We conducted active surveillance of high-risk contacts via text messages or telephone calls until 10 days after their last unprotected exposure. For contacts managed by Barwon South West Public Health Unit, active surveillance was conducted three times per week. We provided all contacts with educational material about avian influenza, and those with ongoing exposure were also given a personal protective equipment factsheet. Barwon South West Public Health Unit arranged a translation of educational material and surveillance text messages into Indonesian for 19 contacts.

#### Suspected cases management

We managed all contacts who reported symptoms as suspected cases, referring them for urgent testing and assessment. We recommended that suspected cases receive 75 mg of oseltamivir twice per day for 5 days. Clinicians collected a nasopharyngeal swab, which was sent to the Victorian Infectious Diseases Reference Laboratory for testing and typing. We advised suspected cases to isolate until a negative result was obtained, and to seek medical review if their symptoms worsened.

#### Human sample testing

Staff at the Victorian Infectious Diseases Reference Laboratory tested clinical samples for potential high-pathogenicity avian influenza by real-time, reverse transcriptase polymerase chain reaction (PCR) under potential laboratory hazard conditions. Staff performed manual extraction of ribonucleic acid (RNA) samples in preparation for PCR while wearing personal protective equipment (in case of airborne pathogens) and using a QiaAmp viral RNA extraction kit (Qiagen, Hilden, Germany).

Laboratory staff synthesized complementary deoxyribonucleic acid (cDNA) using a random hexamer synthesis kit from Meridian Bioline (London, United Kingdom of Great Britain and Northern Ireland); inoculated cDNA templates into separate PrecisionFAST qPCR mastermixes (Primerdesign Ltd, Eastleigh, United Kingdom) with in-house-designed primers and probes (Bioneer Pacific, Kew East, Australia) for influenza A (matrix gene), H5 and H7 real-time PCR;^27^ performed real-time PCR using the ThermoFischer ABI 7500 thermocycler (ThermoFisher Scientific, Waltham, USA) using fluorescence detection up to 45 cycles; and analysed data using Applied Biosystems sequence detection software (ThermoFisher Scientific).

For positive influenza A samples that tested negative for H5 or H7, staff performed multiplex real-time PCR on the cDNA to detect haemagglutinin 1 + 3 genes (H1 + H3) to identify seasonal human influenza. For negative influenza A samples, staff subjected cDNA to a respiratory multiplex and severe acute respiratory syndrome coronavirus 2 (SARS-CoV-2) real-time and conventional PCR using the ThermoFisher 7500 thermocycler.^28^

#### Infection prevention and control

We recommended personal protective equipment for people working on infected premises, including eye protection, N95 mask or equivalent, gloves, overalls and shoe covers. The incident management team developed personal protective equipment resources and provided on-site support through infection prevention and control nurse consultant site visits. personal protective equipment breaches at infected premises during depopulation and decontamination processes required escalation to the relevant public health unit for risk assessment.

### Ethical considerations

Ethics approval was granted by Barwon Health Human Research Ethics Committee (project no. 24/148). The Department of Health, State Government of Victoria provided data obtained via their Public Health Events Surveillance System. We collected and accessed data in accordance with the Public health and wellbeing act 2008 (Victoria).

## Results

### Contacts and cases

We identified a total of 212 contacts, 165 and 47 of which we classified as high and low risk, respectively (Table 1). A geographical distribution of the contacts is available in the online repository.^20^

Contacts resided across 25 Victorian local government areas, with the highest numbers in Greater Geelong (76), Golden Plains (50), Corangamite (20) and Greater Dandenong (15). Country of birth was recorded for 121 contacts (57.1%). Of these, 66 (54.5%) were born in Australia and 55 (45.5%) overseas. The most reported overseas countries of birth were Vanuatu (10), the Philippines (nine), New Zealand (six) and Bhutan (five).

A total of 172 contacts (all high-risk contacts and suspected cases) were eligible for free antiviral medication and seasonal influenza vaccination. Based on information received from local health services and clinics, 19.2% (33/172) received antiviral medication and 27.3% (47/172) received the seasonal influenza vaccine.

**Fig. 1:**
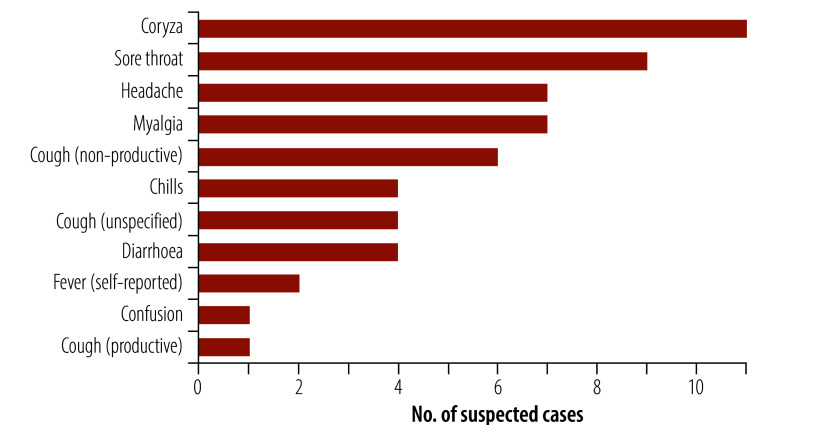
Frequency of symptoms reported by people meeting the suspected case definition who were tested for avian influenza virus, Victoria, Australia, 2024

Note: 20 suspected cases were identified.

We identified a total of 20 suspected cases, associated with six of the eight infected premises. All returned negative test results for influenza A, and there were no probable or confirmed human avian influenza cases. We provide further details of the suspected cases in Table 2 and Fig. 1.

**Table 2:** Infected premises identifier and properties of suspected human cases of avian influenza virus, Victoria, Australia, 2024

Suspected case	Infected premises identifier	Risk category	Laboratory results	Received antivirals	Received influenza vaccination
1	IP1	High	Influenza A negative	Yes	No
2	IP1	High	Influenza A negative	No	No
3	IP1	High	Influenza A negative	Yes	No
4	IP1	Low	Influenza A negative; COVID-19 positive	No	Yes
5	IP1	High	Influenza A negative	Yes	No
6	IP1	High	Influenza A negative	Yes	No
7	IP1, IP2	Low	Influenza A negative; picornavirus positive	No	No
8	IP1	High	Influenza A negative	Yes	No
9	IP1, IP2	High	Influenza A negative	Yes	Yes
10	IP1	Low	Influenza A negative (COVID-19 rapid antigen test positive)	Yes	No
11	IP1	Unknown	Influenza A negative; COVID-19 positive	No	Yes (before outbreak)
12	IP2	High	Influenza A negative	Yes	No
13	IP2	High	Influenza A negative	Yes	No
14	IP3a	High	Influenza A negative	Yes	Yes
15	IP3a, IP5	High	Influenza A negative	Yes	No
16	IP3b	High	Influenza A negative	No	Yes
17	IP3b	High	Influenza A negative	No	Yes
18	IP6	Low	Influenza A negative	Yes	No
19	IP7	High	Influenza A negative	Yes	Yes
20	IP7	High	Influenza A negative	Yes	No

COVID-19: coronavirus disease 2019.

### Other findings

During initial interviews with contacts from identified infected premises, we learnt of poor compliance with personal protective equipment among contacts still working on infected premises; we addressed this through the provision of personal protective equipment resources and site visits from infection prevention and control personnel. We also identified a psychological burden associated with the outbreak; concerns expressed by contacts included animal welfare, financial stress and job security. In response, Western Victoria Primary Health Network developed an information leaflet with a list of local mental health services available to those impacted by the outbreak.

In total, 1.3 million birds were killed and disposed of via deep burial on one of the affected farms. Agriculture Victoria provided compensation to producers if birds died from high-pathogenicity H7 or were destroyed under section 62 of the Livestock disease control act 1994. Payment was not available for losses that were consequential to the disease control effort (e.g. movement restrictions limiting sales). A total of 75.49 million Australian dollars was committed on 16 July 2024 by the National Management Group to address the high-pathogenicity H7 responses in Victoria.^29^

## Discussion

Our report describes the readiness and response procedures that were tested during the largest known Australian outbreak of avian influenza among poultry. As well as the eight identified infected premises in Victoria in June 2024, unrelated outbreaks of high-pathogenicity H7N8 were reported in six properties in New South Wales and two properties in the Australian Capital Territory.^19^

With the increasing global threat of high-pathogenicity H5N1, experts have called for a One Health approach to the management of avian influenza, drawing on expertise from the environmental, animal and human health sectors.^30^ This approach is illustrated by the World Organisation for Animal Health working in partnership with the Food and Agriculture Organization of the United Nations (FAO) and the World Health Organization to monitor zoonotic diseases. FAO is strengthening the capacity of low- and middle-income countries through the implementation of surveillance plans and community education, as well as actively supporting countries during outbreaks.^31^^,^^32^ Our rapid, coordinated, multi-agency response was a success, with key elements including early detection; robust biosecurity measures; and collaboration between local and reference laboratories, primary care facilities and public health authorities. These partnerships enabled prompt identification of high-risk contacts, accessible diagnosis, frontline care and crucial information sharing. 

Improving public awareness, health-care access and education about antivirals and vaccines are important; however, an efficient surveillance system to detect early warning signs of an outbreak and effective prevention strategies are key priorities for low- and middle-income settings. Strict biosecurity measures, good hygiene practices, relevant measures to minimize poultry contact with wild birds, and early reporting of bird illnesses or deaths are essential to control avian influenza.^31^

No human cases were detected among exposed individuals during this outbreak of avian influenza virus in poultry; however, this lack of transmission contributes to our understanding of the risk to humans from the particular strains observed in these outbreaks.

Certain aspects of our response were suboptimal, including the low uptake of antivirals and vaccination, and poor adherence to personal protective equipment guidelines. Barwon South West Public Health Unit are currently surveying identified contacts to explore barriers to vaccination and antiviral uptake. Several of our identified contacts were born overseas or did not speak English fluently. Of those born overseas, many were not enrolled in Medicare (Australia’s universal health insurance scheme), which added challenges in terms of medical assessment and testing. This finding emphasizes the importance of the public health units (i) obtaining demographic information from premises owners early in the outbreak investigation to facilitate prompt translation of resources; and (ii) having clear procedures in place with local health services to facilitate medical assessment and testing for those not enrolled in Medicare. Similar challenges have been identified in the USA during their response to H5N1 on both poultry and dairy farms, reflecting the difficulties faced in real-world implementation of outbreak responses.^33^

An additional issue negatively affecting our response was that many contacts continued to work to depopulate the farms and were therefore unable to attend health services during business hours. Accessing transport was another barrier, although public health units attempted to combat this problem by arranging delivery of antivirals for some contacts. Given the challenges observed during this outbreak, annual free-of-charge seasonal influenza vaccination for poultry workers and piggery workers could enhance pandemic preparedness. 

We focused contact and suspected case management efforts in the Meredith and Terang regions, in which the outbreaks were localized. However, future outbreak responses and pandemic preparedness planning should consider the geographical spread of the residence locations of contacts (in this case, across several local government areas).

Because telephone interviews with contacts were resource intensive for public health units, alternative methods of contact management, for example electronic surveys, could be considered for future outbreaks. However, interviews allowed for the early identification of key issues such as poor personal protective equipment compliance and the need for mental health support, allowing the response to be adapted and improved.

Our study has highlighted the need for improved preparedness, including updated guidelines. An updated version of the Communicable Diseases Network Australia series of national guidelines for avian influenza in humans was published shortly after the avian influenza poultry outbreaks described here, incorporating our findings.^34^

Looking ahead, governments, agricultural industries and health workers must strengthen preparedness and response strategies across national, state, regional and local levels.^5^ These strengthened strategies involve improving surveillance, fostering collaboration and addressing preventive health gaps. Our successful response provides an opportunity for key agencies to reflect on and review current protocols and resources, and to improve the response to future zoonotic disease outbreaks, including those with pandemic potential. We anticipate that elements of our response, particularly the surveillance and tracing activities led by Agriculture Victoria, could be adopted in other settings.
